# Supplementary material for: In-plane staging in lithium-ion intercalation of bilayer graphene
Source: Nat Commun. 2024 Aug 13;15:6933. doi: 10.1038/s41467-024-51196-x (PMC11322308; doi:10.1038/s41467-024-51196-x)
Supplement: Supplementary file 1 — Supplementary Information [file 41467_2024_51196_MOESM1_ESM.pdf]

Supplementary Information

In-plane staging in lithium-ion intercalation of bilayer graphene

Thomas Astles, James G. McHugh, Rui Zhang, Qian Guo, Madeleine Howe, Zefei Wu, Kornelia Indykiewicz, Alex Summerfield, Zachary A.H. Goodwin, Sergey Slizovskiy, Daniil Domaretskiy, Andre K. Geim, Vladimir Falko, Irina V. Grigorieva

1. Supplementary Methods.

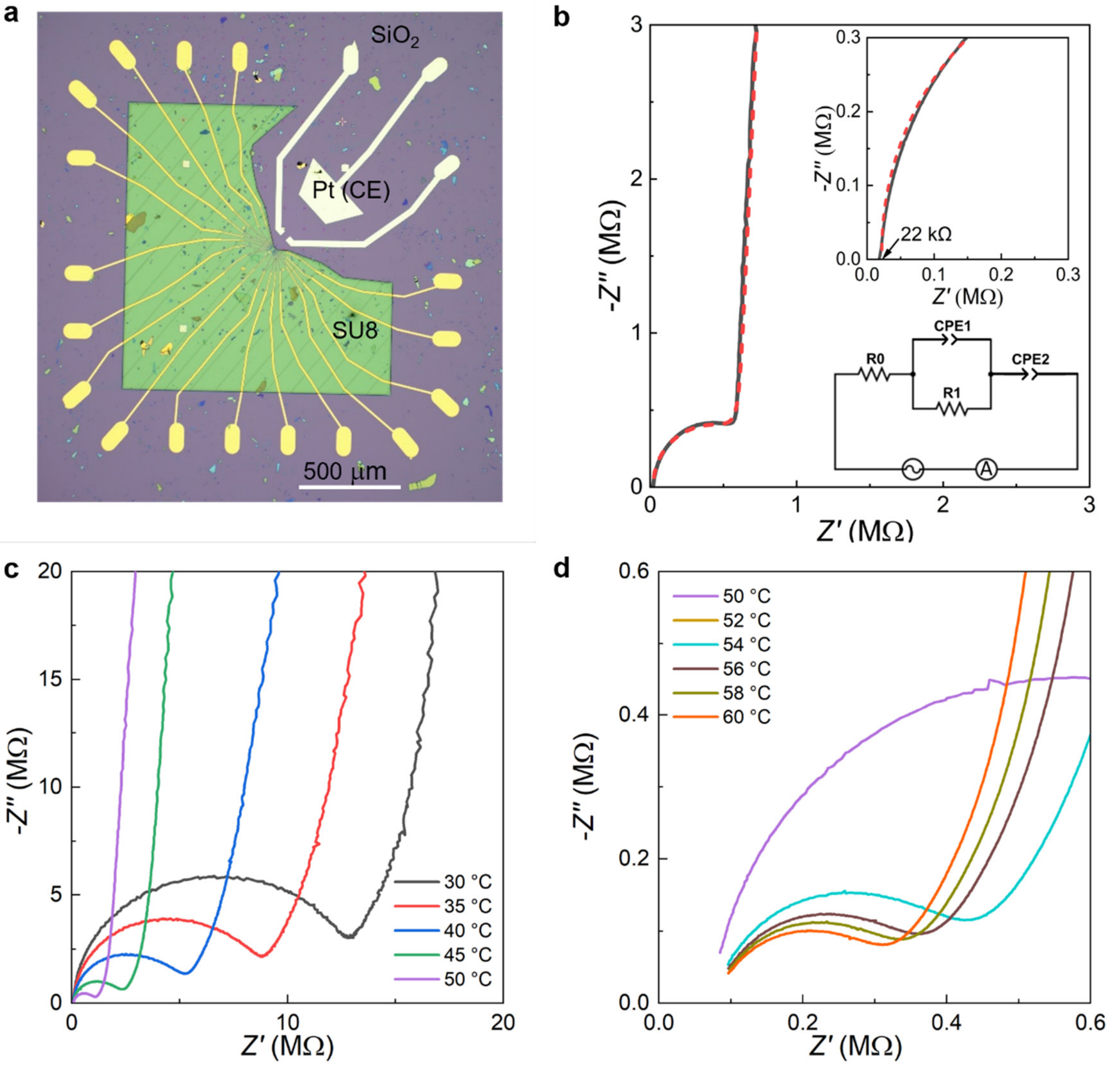

Supplementary Figure 1 | Device overview and impedance spectroscopy characterization. (a) Optical image of the device assembly showing Pt counter- and pseudo-reference electrodes (white) and the protective SU8 layer (green). **(b)** Nyquist plot of the device impedance measured between the Pt counter electrode and BLG at T =57 °C (black solid line) and the equivalent circuit fit obtained using ZView® software (red dashed line). The inset shows a zoom of the high-frequency part indicating the internal resistance of the device, $R_0 \approx 22$ kΩ. Bottom right inset: equivalent circuit. **(c,d)** Nyquist plots of the electrolyte impedance measured between the counter- and pseudo-reference Pt electrodes at different temperatures, see legends.

1.1. Intercalation and transport measurements

A schematic of the on-chip electrochemical cell used in our experiments is shown in Fig. 1a (main text) and the image of a finished device in Supplementary Fig. 1a. To achieve intercalation, the gate voltage V_g was swept from 0 to -7V (or a lower value, as described in the main text) at a constant rate of 10 mV·s^{-1}. For deintercalation, V_g was returned to 0V at the same rate. We have used Pt both for the counter- and the pseudo-reference electrodes; the source of Li ions in our electrochemical cell is the LiTFSI salt in the electrolyte. The role of the counter electrode is to create a potential drop at the interface with BLG, such that the chemical potential of Li in C_xLiC_x becomes lower than the chemical potential of 'free' lithium in the electrolyte (with an unknown chemical potential). The threshold potential for intercalation in this case does not correspond to any thermodynamic value (as in the case of Li/Li$^+$ electrode) and was determined experimentally for each device. To compare the experimental results with our DFT calculations of the free energy, we defined a reference potential as $\mu_{\text{ref}} = eV_{\text{ref}} = \mu + \Delta\mu$, where V_{ref} is the measured pseudo-potential and $\Delta\mu = 0$ corresponds to the start of intercalation (sharp peak in the device resistance, see below). This allowed us to determine μ experimentally as $\mu \approx eV_{\text{ref}}$ at $\Delta\mu = 0$. For example, for a device in Supplementary Fig. 7a below we found $V_{\text{ref}} \approx 2.9$ V, that is, $\mu_{\text{ref}} = \mu \approx 2.9$ eV. The applied V_g in this case was -4.5 V; the difference between V_g and V_{ref} is due to a voltage drop at the counter electrode.

We note that, although $V_g = -7$ V nominally exceeds the electrochemical stability window for PEO-LiTFSI electrolyte [1], we found that a large fraction (30 to 40%) of the applied 7V potential difference falls at the Pt counter electrode, where no faradaic reactions are expected. The potential drop at the graphene interface (as found from our measurements of the potential difference between the Pt pseudo-reference and graphene) is then < 4.5V, comparable or less than the reported stability window ~4.5 V vs Li/Li$^+$ [1]. The additional voltage drop due to the relatively high internal resistance of our devices ($R_0 \approx 20$ kΩ, Supplementary Fig. 1b) was always negligible compared to the applied gate voltage, <0.2 mV, due to very small anodic/cathodic currents in our experiments, $I < 10$ nA. For the number of cycles used in our study (<20) we did not see significant changes in electrolyte conductivity or in the internal device resistance after cycling, as confirmed by repeated impedance spectroscopy measurements. We also did not see any visible evidence of the electrolyte movement when inspecting the devices before and after intercalation.

A magnetic field $B = \pm 330$ mT was provided by a permanent magnet positioned immediately above the device within the glovebox. The density of Li ions, n_{Li}, at each stage of intercalation was found from the carrier density in the BLG, n, measured at certain points during intercalation and at the end of each intercalation (deintercalation) half-cycle. Here $n = B \cdot I/(e \cdot V_{\text{Hall}})$ was determined from the Hall voltage measured at ± 330 mT, where $V_{\text{Hall}} = [(V_{xy}(B) - V_{xy}(-B)]/2$, I = 1 μA is the applied current and e the electron charge. BLG in all our as-fabricated devices was initially p-doped (carrier density $n_0 \approx$ -1.2 x10^{13} cm^{-2}, where the minus sign is used to indicate hole doping). Such level of p-doping is typical for unencapsulated graphene in contact with polymers [2,3] and, in our case, was probably caused by fabrication residues and/or SU-8 layer [2]. Taking into account the initial p-doping, the density of intercalated Li ions was calculated as $n_{\text{Li}} = (n + |n_0|)/0.9$, where $|n_0|$ is the carrier density in the bilayer at the start of intercalation ($t = 0$) and it is assumed that each Li ion donates 0.9e to the BLG host (see main text). We note that in the literature the DFT charge transfer value for Li in graphite/graphene varies from 0.85 to 0.88 to 0.9e$^-$; we are using a rounded value of 0.9e$^-$ throughout. Using a lower value of 0.88e$^-$ would result in just 1-2% difference in n_{Li}, which is below our experimental error and did not affect the inferred Li ion configurations or other conclusions. To estimate n_{Li} for stages II and III, which were too short in duration to measure V_{xy} at $\pm B$ (open symbols in Fig. 3a), we used the assumption of a constant scattering time τ in $\rho_{xx} = m^*/(ne^2\tau)$ for each stage of intercalation, see discussion in the main text (m^* is the electron mass). The carrier density was then

estimated as $n \approx A/\rho_{xx}$, where $A = m^*/e^2\tau$ was assumed to be constant and found as $A \approx \rho_{xx} n_{meas}$ for a given stage using both resistance and Hall measurements as described above.

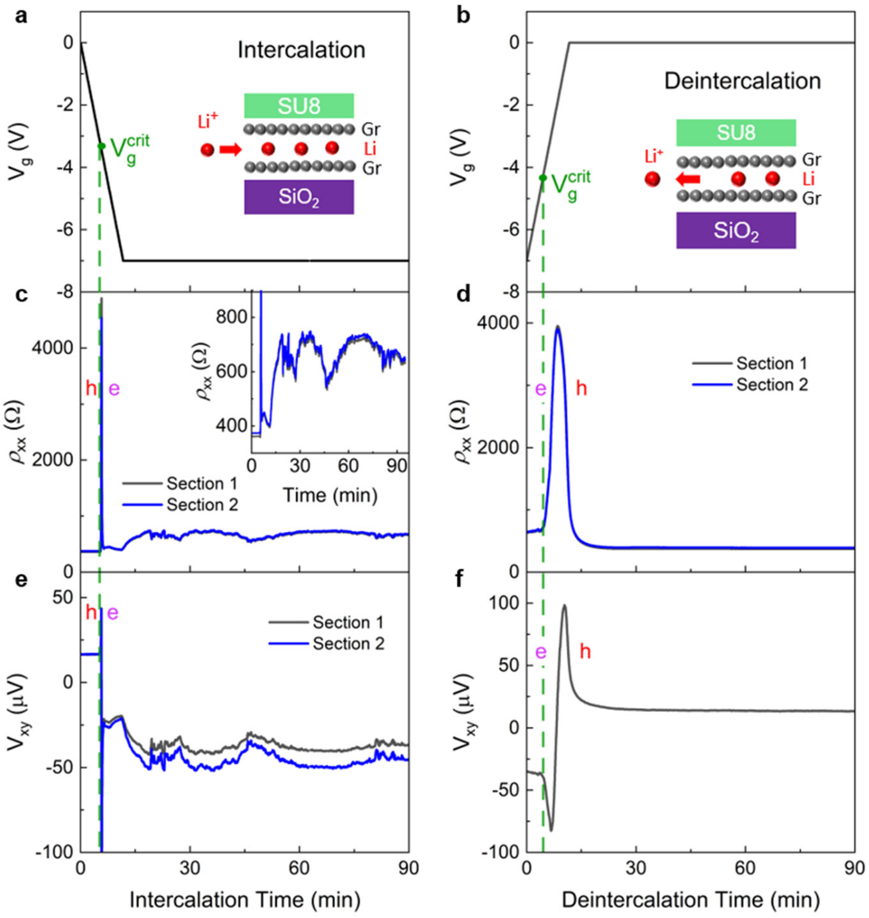

Supplementary Figure 2 | First intercalation-deintercalation cycle. Evolution of the longitudinal resistance and Hall voltage of BLG during intercalation (c,e) and deintercalation (d,f). Blue and black curves in (c,e) show ρ_{xx} and V_{xy} measured at two different sections of the device, separated by ~10 μm. Top panels (a,b) show the corresponding sweep of the gate voltage V_g and indicate its critical value corresponding to the start of intercalation (deintercalation).

For completeness, Supplementary Figures 2&3a show $\rho_{xx}(t)$ and $V_{xy}(t)$ for the 1st intercalation-deintercalation cycle and $\rho_{xx}(t)$ for all 14 intercalation cycles for device A, respectively. In the 1st cycle (Supplementary Fig. 2), as the gate voltage V_g was swept from 0 to -7V, ρ_{xx} changed from a steady value of ~350 Ω in the initial p-doped state to ~420 Ω and $n \approx 8 \times 10^{12}$ cm^{-2} in the intercalated state, with the change of graphene's polarity occurring over ~1 min, at $V_g \approx -3$V. This indicated a fast entry of a large number of Li ions corresponding to interionic distances ~2.5 nm, or one Li per 170 carbon atoms. However, after a few minutes ρ_{xx} started to increase and underwent significant fluctuations, even though all external factors remain the same: V_g kept at -7 V, temperature at 57°C, etc. Furthermore, the fluctuations that were much larger than the measurement noise (~1Ω) occurred simultaneously across the entire device (cf. ρ_{xx} and V_{xy} for two different pairs of contacts in Supplementary Fig. 2c,e). In contrast to the intercalation behavior, deintercalation proceeded smoothly and was slower (Supplementary Fig. 2d,f), with the bilayer returning to its initial p-doped state after approximately 30 min for this first cycle. In the 2nd cycle, ρ_{xx} in the intercalated state remained at a more constant value but fluctuations – and therefore instabilities in the amount of

intercalated Li ions – remained. The maximum Li ion density at the end of intercalation in this cycle was $n_{\mathrm{Li}} \approx$ 5.3 x10^{13} cm^{-2}, or one Li ion per ~140 carbon atoms, giving an average composition ~$C_{70}LiC_{70}$, that is, a very dilute and likely disordered intercalation state. It is possible that intercalation in the initial one or two cycles was affected by formation of a solid-electrolyte interphase (SEI) and it may have contributed to the observed changes (in addition to bilayer expansion and formation of AB/BA boundaries discussed in the main text). Importantly, SEI formed at negative potentials is porous and not expected to prevent intercalation [1,4].

The 3rd cycle was characterized by an almost constant $\rho_{\mathrm{xx}} \sim 250\ \Omega$ maintained over >1h and much smaller resistance fluctuations compared to the 2nd cycle. First signs of stable Li configurations and the development of staging appeared from the 4th cycle onwards as described in the main text.

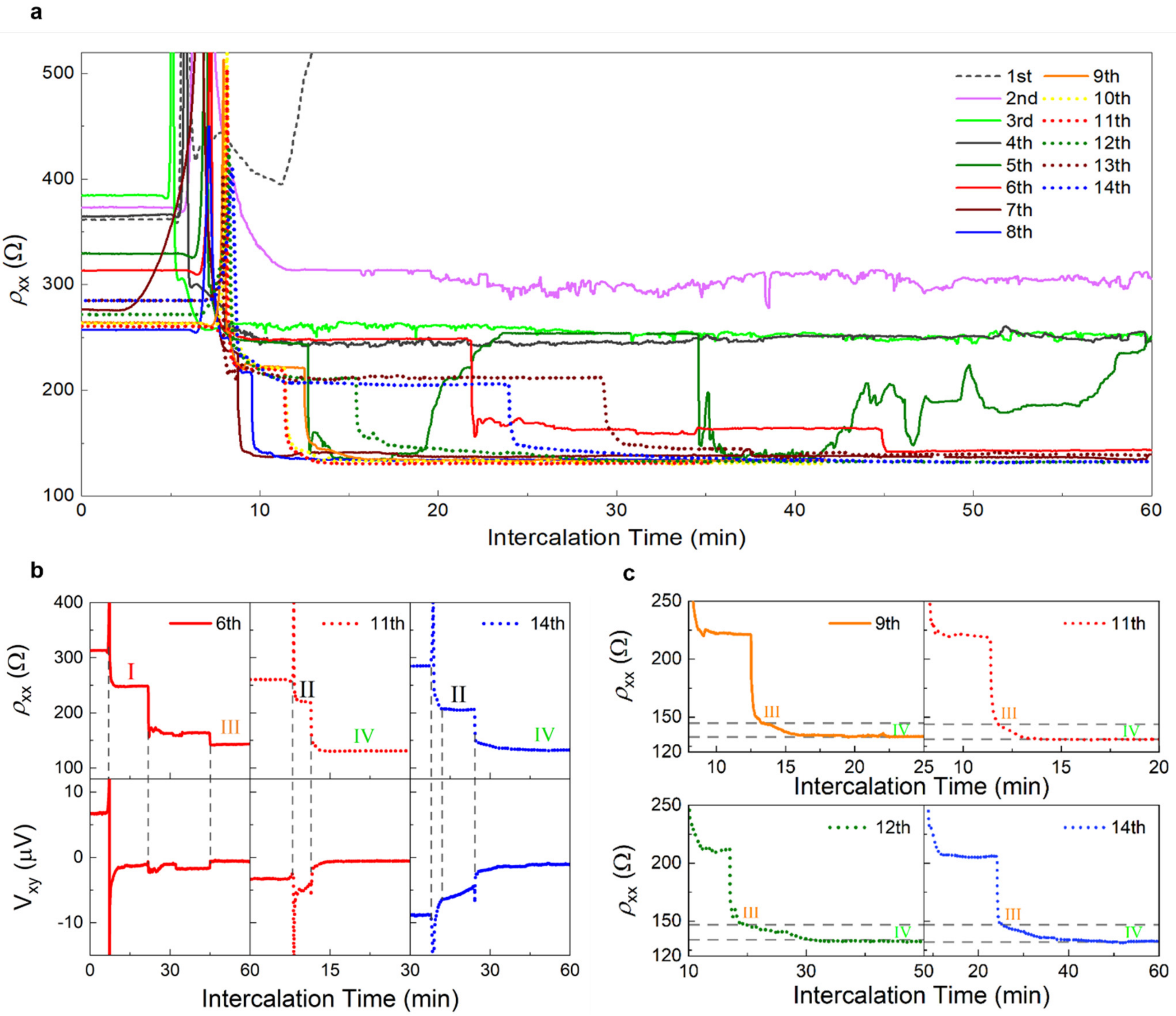

Supplementary Figure 3 | Intercalation evolution and developing staging. (a) Evolution of the longitudinal resistance during the first hour in 14 consecutive intercalation cycles (see legends for the cycle numbers). The resistance in the 1st cycle (black dashed curve, not shown after 10 min) remains > 500 Ω and is shown separately in Supplementary Fig. 2a. **(b)** Simultaneous measurements of the longitudinal resistance ρ_{xx} and Hall voltage V_{xy} during intercalation. The top panels show evolution of ρ_{xx} for the selected cycles and the bottom panels show corresponding V_{xy}. The measurements were done under a constant magnetic field of 330 mT. **(c)** Gradual changes in ρ_{xx} signifying the transition from stage III to stage IV (see the main text). Shown are several representative cycles. The dashed lines indicate the ρ_{xx} values for intercalation stages III and IV. All the data are from device A.

Supplementary Fig. 4b demonstrates that achieving the high-density intercalated state (stage IV) did not depend on the magnitude of V_g, as long as it exceeded the critical value (typically, $V_g \approx -3.5$ V in our experiment). To this end, in one of the intercalation cycles for device B (Supplementary Fig. 4) we used $V_g = -3.8$ V, well below $V_g = -7$ V used in other measurements. This resulted in a much longer time needed for the transition from stage I to stage IV but did not affect the final result. A later increase of V_g to -7 V did not have any effect on the intercalation process. This finding is consistent with the known effect of the overpotential on the interfacial kinetics and Li ion diffusion in graphite [5]: at small overpotentials, such as used in this experiment, the limiting factor for Li ion insertion is likely to be the interfacial charge transfer resistance, rather than the diffusion of ions, resulting in slow kinetics.

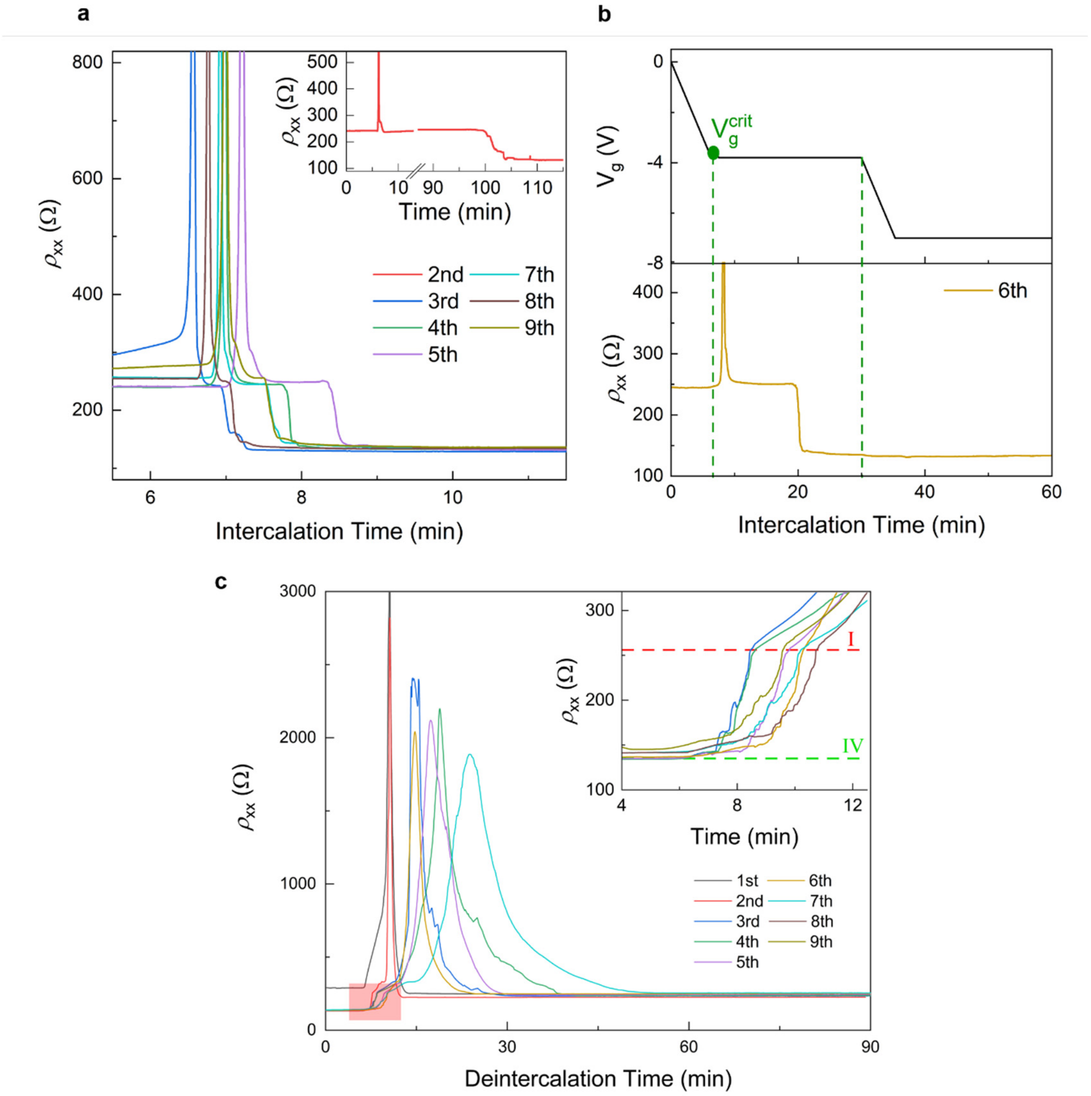

Supplementary Figure 4 | Further examples of intercalation behavior and the effect of the driving (gate) potential. (a) Representative intercalation cycles for device B. Longitudinal resistance values in this device are identical (within experimental accuracy) to those for device A (Fig. 1 in the main text), i.e., $\rho_{xx} = 248 \pm 4$ Ω for stage I and $\rho_{xx} = 133 \pm 3$ Ω for stage IV. Also, similar to device A, the transition to a higher stage occurred only after > 100 min in the 2nd cycle (inset). **(b)** Time evolution of ρ_{xx} at a lower driving voltage $V_g = -3.8$ V (just above the threshold value) as opposed to $V_g = -7$ V used in most measurements. The transition from stage I to stage III/IV is seen to occur at $V_g = -3.8$ V but it takes a significantly longer time for this to happen [cf. panel (a)]. No further changes are seen as V_g is increased to -7 V. **(c)** Representative deintercalation half-cycles for device B (color coded curves). The inset shows a zoom of the ρ_{xx} curves within the pink square in the main panel. Similar to device A, the transition from stage IV to stage I is clearly seen at the beginning of deintercalation, as well as broadening of the resistance peak and a gradual decrease in the maximum value of ρ_{xx} in later cycles.

For completeness, Supplementary Fig. 5 shows additional data for deintercalation of device A and Supplementary Fig. 6 illustrates the reproducibility of the reported in-plane staging for different devices.

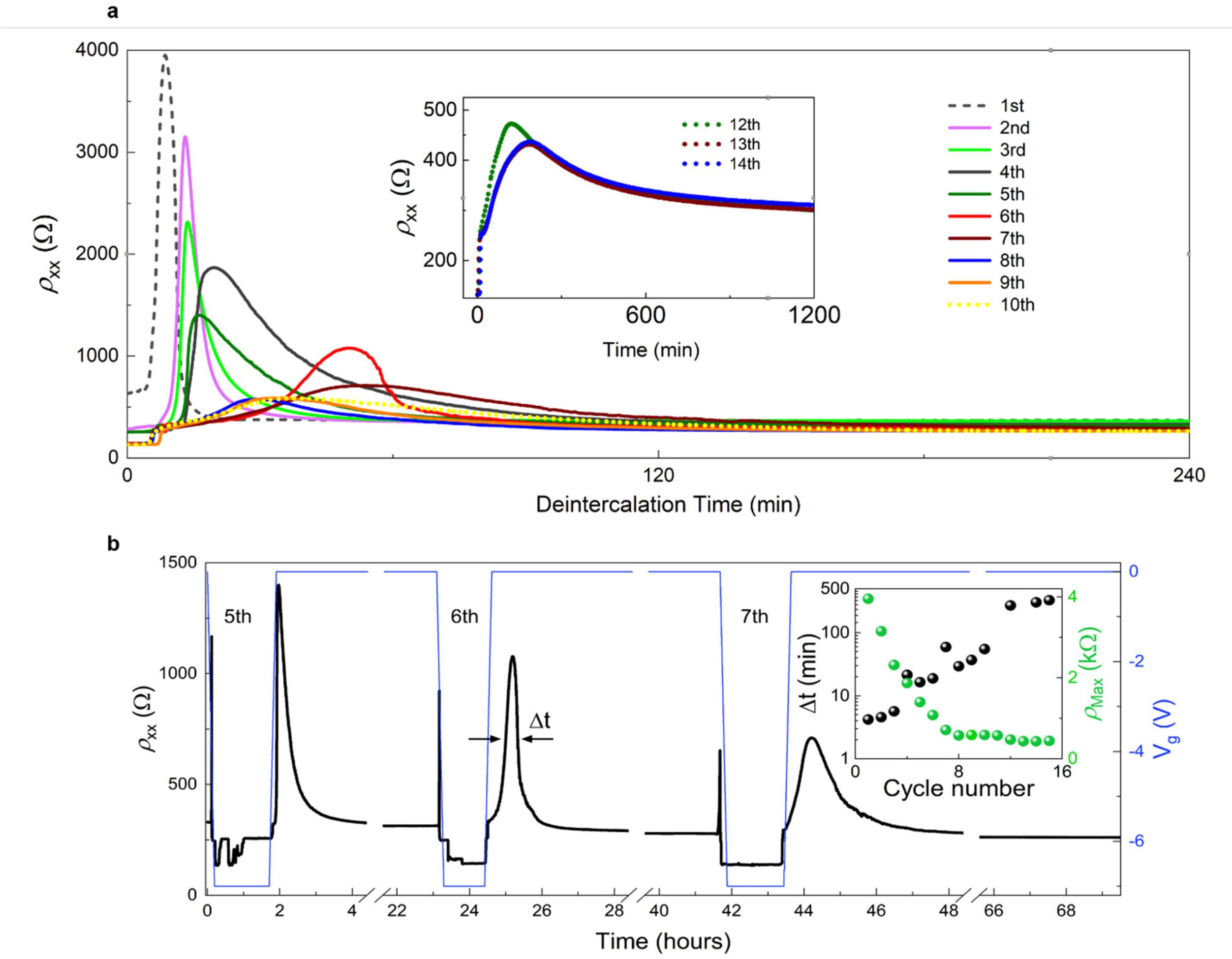

Supplementary Figure 5 | Evolution of the bilayer resistance during deintercalation. (a) Time dependence of ρ_{xx} during deintercalation in all consecutive cycles for device A (see legends for cycle numbers). Same color-coding as for the intercalation curves in Fig. 1d (main text) and Supplementary Fig. 3a. **(b)** Consecutive intercalation-deintercalation cycles (5th, 6th, 7th) illustrating gradually slowing deintercalation dynamics. Blue and black curves show $V_{\mathrm{g}}(t)$ and $\rho_{\mathrm{xx}}(t)$, respectively. *Inset:* Changes in the width and height of the deintercalation resistance peak, Δt and ρ_{max} respectively, with the cycle number. Data for device A.

To assign stoichiometric compositions to intercalation stages, we calculated Li:C ratios (N in $C_N LiC_N$) using the extracted average Li densities n_{Li}: $N = 2/n_{\mathrm{Li}}A$, where $A = 5.23{\cdot}10^{-16}$ cm^2 is the area of a hexagonal unit cell containing 2 C atoms. The values of N calculated in this way for stages I, II, III and IV were 42.0, 37.8, 17.2, 13.9 which were rounded to the nearest integer yielding $C_{42}LiC_{42}$, $C_{38}LiC_{38}$, $C_{18}LiC_{18}$ and $C_{14}LiC_{14}$ (rounding to 18 for stage III ensured Li positions in the centres of C hexagons). Additionally, we have calculated the *expected* n_{Li} for N =42, 38,18, and 14 as $n_{\mathrm{Li}} = 2/N{\cdot}A$ – these are shown as horizontal lines in Fig. 3a in the main text.

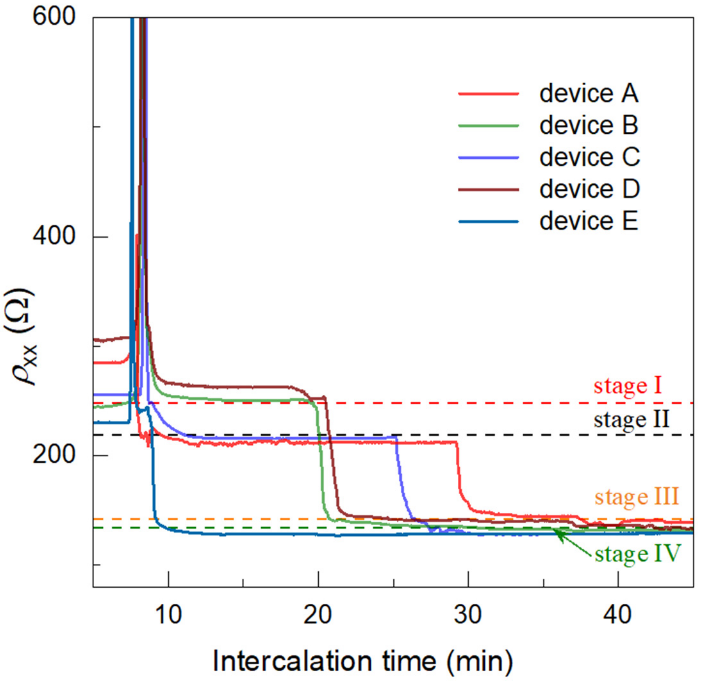

Supplementary Figure 6 | Reproducibility of staging on different devices. Time evolution of ρ_{xx} for all 5 studied devices. Shown are representative (middle) cycles, where staging has already been established. As described in the main text, for some of the devices the intermediate plateaus (stages II and/or III) were missing in any of the studied cycles. Additionally, the time duration of the more dilute stages was sometimes very short (e.g., device E), where the bilayer transitioned very quickly from stage I to stage IV.

1.2. Raman spectroscopy

As an alternative method to monitor intercalation, we used *in operando* Raman spectroscopy. The device of the same design as in Supplementary Fig. 1 was placed in a sealed custom-made chamber with an optical (quartz) window. A thin layer of the electrolyte was deposited prior to sealing in an Ar-filled glovebox. The gate voltage V_g was swept in small steps while continuously measuring the longitudinal resistance, and a Raman spectrum taken at the end of each step. We used a commercial WITec Raman spectrometer with 514 nm excitation laser, and used three 80s acquisitions per spectrum. Representative spectra before, during and after intercalation are shown in Fig. 5. As noted in the main text, no D peak could be detected either before, during or after intercalation. Nevertheless, it is still possible that a very small D peak (below the noise level) was present, so we estimated the upper limit on the defect density from the corresponding (implied) $I_{\rm D}/I_{\rm G}$ ratio. In deintercalated state, assuming $I_{\rm D} \leq 30$ (noise level), we obtained $I_{\rm D}/I_{\rm G} < 0.02$ and the upper limit on the defect density $n_{\rm D} < 7.5 \times 10^9 \times E_{\rm L}^4 \times I_{\rm D}/I_{\rm G} \approx 5{\cdot}10^9$ cm^{-2} (here $E_{\rm L} = 2.4$ eV is the laser excitation energy) [6].

2. Supplementary Notes

2.1. *Ab-initio* thermodynamic analysis of Li intercalation into BLG

DFT calculations were performed using Quantum ESPRESSO code [7,8]. The Li to C ratio was varied by inserting a single Li atom into BLG supercells of different dimensions, through choice of different crystallographic supercell lattice vectors. A spacing of 20 Å between repeated images in the out-of-plane direction was applied to avoid interactions between these images. GBRV ultrasoft pseudopotentials were

used to approximate the effect of core electrons [9], parameterized using the Perdew-Burke-Ernzerhof (PBE) Generalized Gradient Approximation (GGA) of the exchange-correlation functional [10]. The Brillouin zone was sampled using a regular Monkhorst-Pack k-point grid of $28 \times 28 \times 1$ in a BLG unit cell [11], and calculations in larger cells were fixed at the same k-point density. The optB88-vdW DFT functional was used to model van der Waals interactions [12]. Coulomb interactions in the out-of-plane direction were truncated to avoid spurious long-range interactions. This was found to increase the calculated DFT energies, both for AA and AB stacking of Li-intercalated bilayer graphene, see Supplementary Fig. 8a.

To extract the intercalation energy per Li ion from DFT data we used the expression

$$E_{\mathrm{int}} = \frac{(E_{\mathrm{cell}} - N_{\mathrm{C}}\epsilon_{\mathrm{BLG}})}{N_{\mathrm{Li}}} - \mu_{\mathrm{ref}}\,, \quad (1)$$

where E_{cell} is the energy of a fully relaxed DFT cell with a given Li density (ratio of the number of Li ions N_{C} to the number of carbon atoms N_{Li}), ϵ_{BLG} is the energy per carbon atom of AB-stacked bilayer graphene, and μ_{ref} is a reference chemical potential of intercalated Li ions with respect to 'free' lithium. In previous computational studies, the reference chemical potential (μ_{ref}) was often chosen with respect to metallic Li [13] or an isolated Li atom [14]. As in our experiment 'free' lithium is in the form of mobile ions in LiFTSI electrolyte (with an unknown chemical potential), we choose $\mu_{\mathrm{ref}} = \mu + \Delta\mu$ and calibrate μ by calculating the Gibbs free energy such that intercalation of Li ions is energetically disfavored for all Li densities, i.e., such that $\Delta\mu$=0 (corresponding Gibbs free energy as a function of Li density is shown in the inset of Supplementary Fig. 8b).

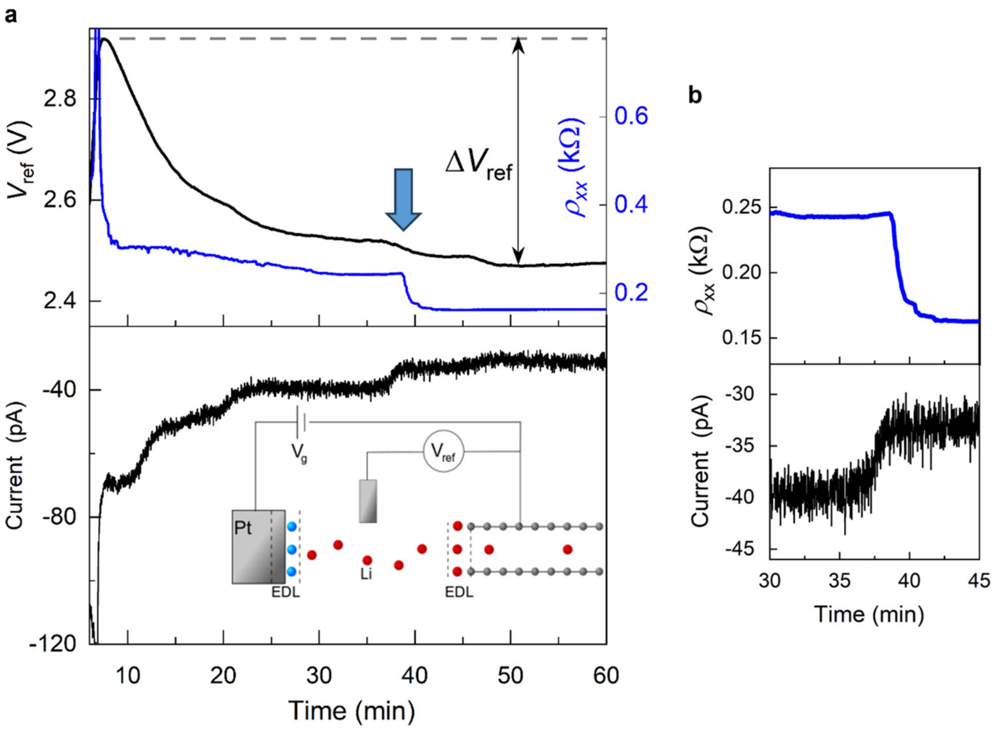

Supplementary Figure 7 | Experimental determination of the difference in chemical potentials of Li ions in the source (electrolyte) and the intercalated bilayer. (a) *Top panel*: Simultaneous measurements of the pseudo-reference potential V_{ref} (black) and the longitudinal resistance ρ_{xx} of the device (blue). The difference in chemical potentials is determined as $\Delta\mu = e\Delta V_{\mathrm{ref}}$, where ΔV_{ref} is the drop in reference potential between the start of intercalation (sharp peak in ρ_{xx}) and the fully intercalated state, as indicated in the figure. *Bottom panel*: Corresponding current through the electrolyte. *Inset:* Measurements schematic. EDL refers to the electric double layer at the solid-electrolyte interface. **(b)** Zoom of the ρ_{xx} step and the corresponding step in cathodic current used to estimate the charge transfer corresponding to the transition from stage I to stage III (see text).

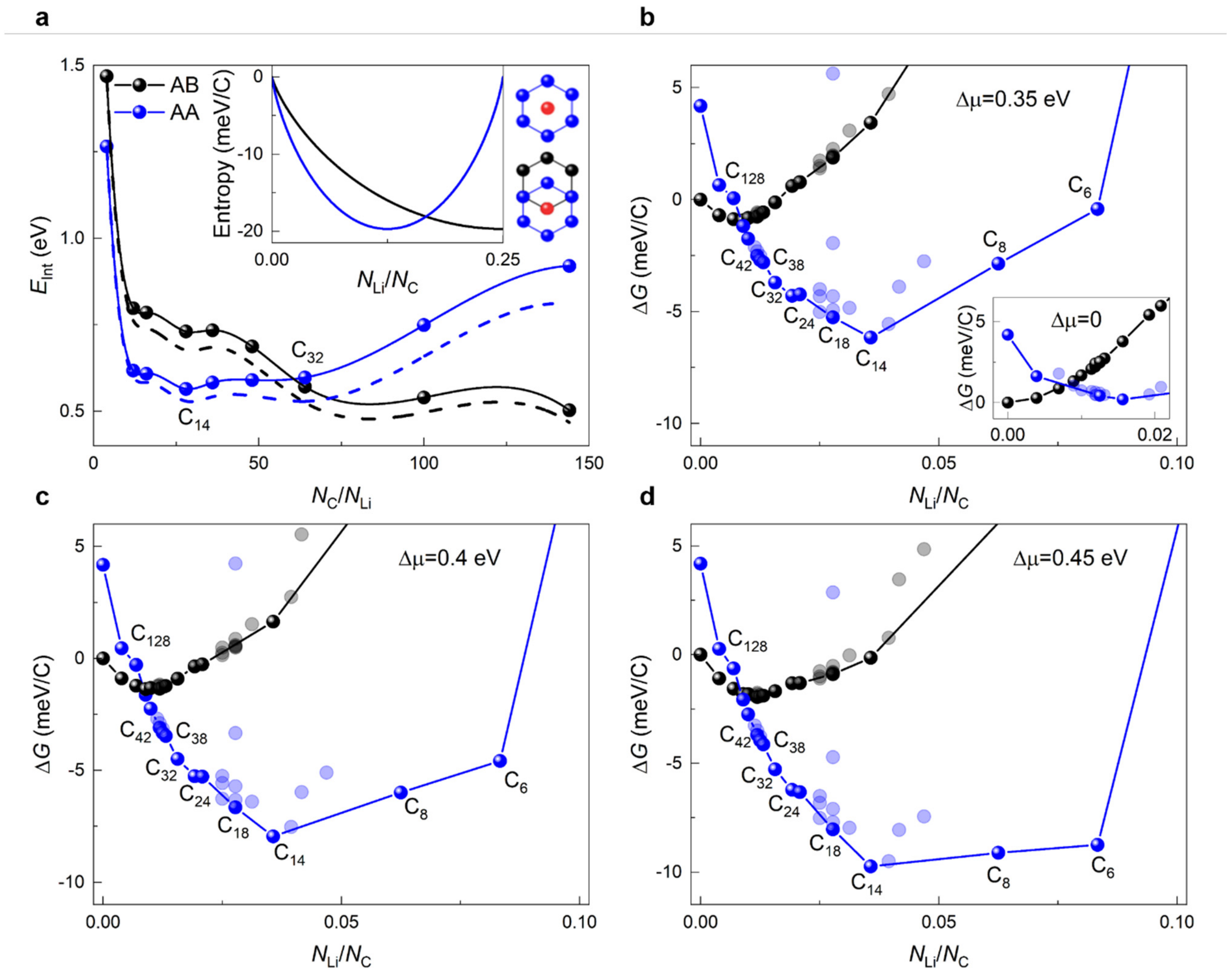

Supplementary Figure 8 | DFT intercalation energy and Gibbs free energy for reference chemical potentials of Li corresponding to the experiment. (a) Main panel: Intercalation energy vs the ratio of the number of carbon atoms N_{C} to the number of lithium ions N_{Li} for AB- and AA stacked bilayer graphene. Local minima in E_{int} correspond to $C_{14}LiC_{14}$ stoichiometry. Solid lines show the results of calculations where Coulomb interactions in the out-of-plane direction were truncated to avoid spurious long-range interactions (as appropriate for BLG). Dashed lines show the results without truncation (corresponding to intercalation energy for bulk graphite). *Inset*: Entropy contribution vs Li density for AA and AB stacking, blue and black line, respectively. **(b-d)** Gibbs free energy vs lithium density at values of $\Delta\mu$ covering the range of its experimental uncertainty (see text). In experiment, $\Delta\mu$ was found to be 0.4±0.02 eV. Labels show *N* for the corresponding $C_N LiC_N$ stoichiometries. Bright blue and black symbols correspond to Li ion configurations with the hexagonal symmetry and light blue/grey to non-hexagonal configurations with non-equidistant Li ion positions. Solid lines connecting data for the hexagonal symmetry are guides to the eye.

Using the above intercalation energy and incorporating entropy terms, the Gibbs free energy (relative to unintercalated state) at different Li densities is

$$\Delta G = \rho E_{\mathrm{int}} - \rho\Delta\mu + k_B T\,[\bar{\rho}\ln(\bar{\rho}) + (1-\bar{\rho})\ln(1-\bar{\rho})], \qquad (2)$$

where $\rho = N_{\mathrm{Li}}/N_{\mathrm{C}}$ gives the density of Li ions, $\bar{\rho} = \frac{\rho N_C}{N_{\mathrm{sites}}}$ accounts for the number of available intercalation sites [15] and $\Delta\mu$ can be related to experimentally measured values of the pseudo-reference potential (Supplementary Fig. 7). In experiment $\Delta\mu = 0$ is equivalent to the pseudo-reference potential, V_{ref}, corresponding to the start of Li ions entry into the bilayer, i.e., to the sharp peak in ρ_{xx}. Accordingly, $\Delta\mu$ in

eq. (2) corresponds to the change of V_{ref} resulting from Li intercalation, $\Delta\mu = e\Delta V_{\text{ref}} = 0.4 \pm 0.02$ eV, see Supplementary Fig. 7. For the temperature fixed at the experimental value $T = 330$ K and $\Delta\mu = 0.4$ eV, the evolution of the Gibbs free energy as a function of Li density (ratio of the number of Li ions to the number of carbon atoms) is shown in Fig. 6 in the main text. To accommodate the experimental range of ΔV_{ref} obtained in different intercalation cycles, Supplementary Fig. 8b-c shows the DFT results for $\Delta\mu = 0.35, 0.4$ and 0.45 eV, with the global minimum in the Gibbs energy remaining at $N = 14$ ($C_{14}LiC_{14}$ composition).

At low Li densities both the intercalation energy E_{int} and the Gibbs energy ΔG are lower for AB stacking, while at higher $N_{\text{Li}}/N_{\text{C}}$ both energies are lower for the AA stacked bilayer. It is notable, however, that for the intercalation energy the crossover between AB and AA stacking occurs at a significantly higher Li to carbon ratio, $C_{32}LiC_{32}$ versus $C_{54}LiC_{54}$ for the Gibbs free energy, compare Supplementary Figs 8a and 8b-c. This is the consequence of a large difference in entropy contributions for the two bilayer stackings, because there are twice the number of available intercalation sites in AB-stacked BLG, compared to AA. The smaller number of available intercalation sites in AA-BLG saturates at a lower Li density and therefore favors AB over AA at low densities, see inset if Supplementary Fig. 8a. At $T = 330$ K, this effect is responsible for pushing the predicted AB-to-AA restacking transition ($\Delta G_{\text{AA}} = \Delta G_{\text{AB}}$) from $C_{32}LiC_{32}$ to approximately $C_{54}LiC_{54}$ (see Supplementary Fig. 8b-d). This result is in agreement with reported experimental observations of a large entropy contribution at low Li densities in intercalated graphite [16].

Both the intercalation energy E_{int} and the Gibbs energy ΔG_{AA} show minima for the $\sqrt{7} \times \sqrt{7}$ superlattice of Li ions ($C_{14}LiC_{14}$ stoichiometry). In the latter case $C_{14}LiC_{14}$ corresponds to the thermodynamic equilibrium between the chemical potential of Li ions in the bilayer and in the electrolyte, $\frac{\partial G_{\text{AA}}}{\partial \rho} = 0$.

2.2. Domain nucleation

While the Li density corresponding to thermodynamic equilibrium is in excellent agreement with experiment, the calculated Li density corresponding to the Gibbs energy crossover from AB and AA stacking appears to be notably lower, $C_{54}LiC_{54}$ as opposed to experimental $C_{42}LiC_{42}$ / $C_{38}LiC_{38}$ where the change of bilayer stacking is inferred from the transition from stage I/II to stage III/IV (Fig. 3a in the main text). This is due to the fact that restacking requires nucleation of AA domains and these have to be of a sufficient size in order to continue to grow over the whole sample, similar to the growth of nanoparticles but in 2D. To estimate the minimum size of nucleating AA domains in an initially AB-stacked bilayer graphene, we approximate the domain energy as the sum of an areal energy density σ associated with restacking of Li-intercalated BLG (energy gain) and an elastic penalty per unit length of the AA-AB domain wall γ:

$$E(r) = -\sigma A(r) + \gamma C(r). \qquad (3)$$

Here A and C are the area and circumference of a circular domain, respectively, and the critical radius for the formation of an AA domain is $r_{\text{c}} = 2\gamma/\sigma$. Here σ = 2.6 meV/Å^2 is the calculated Gibbs energy difference for AB and AA stacking of the Li intercalated bilayer. For the line tension we could assume $\gamma \approx 0.1$ eV/Å as found in literature for AB/BA shear domain walls [17,18]. However, a circular domain wall between AA- and AB-stacked regions requires a constant Burgers vector at the boundary, which leads to significant bending of the wall and creates hydrostatic strain, in addition to the pure shear strain of a simple screw dislocation analyzed in refs [17,18]. To account for this, we have calculated the elastic energy explicitly in LAMMPS [19] using the AIREBO potential for intralayer interactions [20] and DRIP for interlayer adhesion [21]. Fixed AA domains were created inside a graphene bilayer, while all atoms outside of these fixed regions were allowed to relax, after which the total elastic energy was calculated as the difference between individual deformed

monolayers and perfect graphene. The calculated line tension (γ = 0.16 eV/Å) for a 20 nm AA region is notably higher than that of a perfect shear domain wall. To estimate the critical domain size, we have compared the adhesion and elastic energies as a function of domain radius as shown in Supplementary Fig. 9. This yielded $r_c \approx$ 12 nm corresponding to ~480 Li ions per domain.

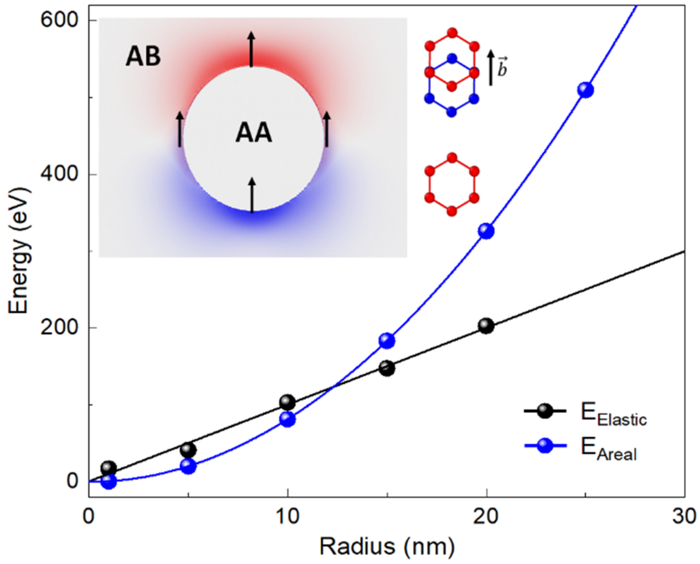

Supplementary Figure 9 | DFT energy contributions to formation of an AA domain in an initially AB-stacked bilayer intercalated with Li. Black symbols: elastic energy as a function of the domain radius. Blue symbols: areal stacking energy. *Inset*: Strain distribution around an AA-stacked domain. Hydrostatic tension and compression alternate at different sides of the domain, resulting from the constant Burgers vector at the surrounding domain walls as shown to the right.

2.3. Lithium ion diffusion

Our experiment suggests significant differences in Li ion diffusivity in AB- and AA stacked bilayers. These differences can be understood by considering the energy of a Li ion as a function of lateral displacement within the BLG. Supplementary Fig. 10a,b shows the energy maps for a Li ion diffusing in AA- and AB-stacked bilayers. These are computed, neglecting relaxation, for the Li ion position in the vertical direction halfway between two rigid graphene layers. Supplementary Fig. 10c,d shows corresponding energy barriers for optimal diffusion paths by one lattice constant, which involves a single jump between adjacent sites for AA stacking and two jumps between energetically equivalent sites for AB stacking. Notably, for AA stacking this barrier is higher due to more significant overlap of Li and C valence electrons.

The computed DFT activation barriers can be related to diffusivity D [22,23]:

$$D = \frac{1}{2d}\Gamma_T \alpha^2,$$

where α is the jumping distance ($\alpha = a$ and $\alpha = a/\sqrt{3}$ for Li ions in AA and AB-stacked BLG, respectively), $d = 2$ is the dimensionality of the diffusion process, and Γ_T is the total frequency of the jumps between adjacent sites. The total frequency is $\Gamma_T = \sum_n \omega_n$, where n is the number of adjacent sites ($n = 3$ for AA- and $n = 6$ for AB stacking), and ω_n is the frequency of a single inter-site diffusion event. The latter is calculated from DFT energy barriers using the Arrhenius equation

$$\omega_n = \nu \exp(-\beta E_{a,n}),$$

with $\beta = 1/k_B T$, $\nu \sim 10^{13}$ Hz the attempt frequency of a single diffusive jump [22], and $E_{a,n}$ the DFT energy barrier. Our DFT calculations yield the energy barriers $E = 0.28$ and 0.07 eV for Li diffusion in AA- and AB-stacked BLG, respectively, with corresponding diffusion constants $D \approx 2.4 \times 10^{-7}$ and 2.6×10^{-4} $cm^2{\cdot}s^{-1}$. These values suggest significantly faster ion diffusion in AB-stacked bilayer, in agreement with experiment.

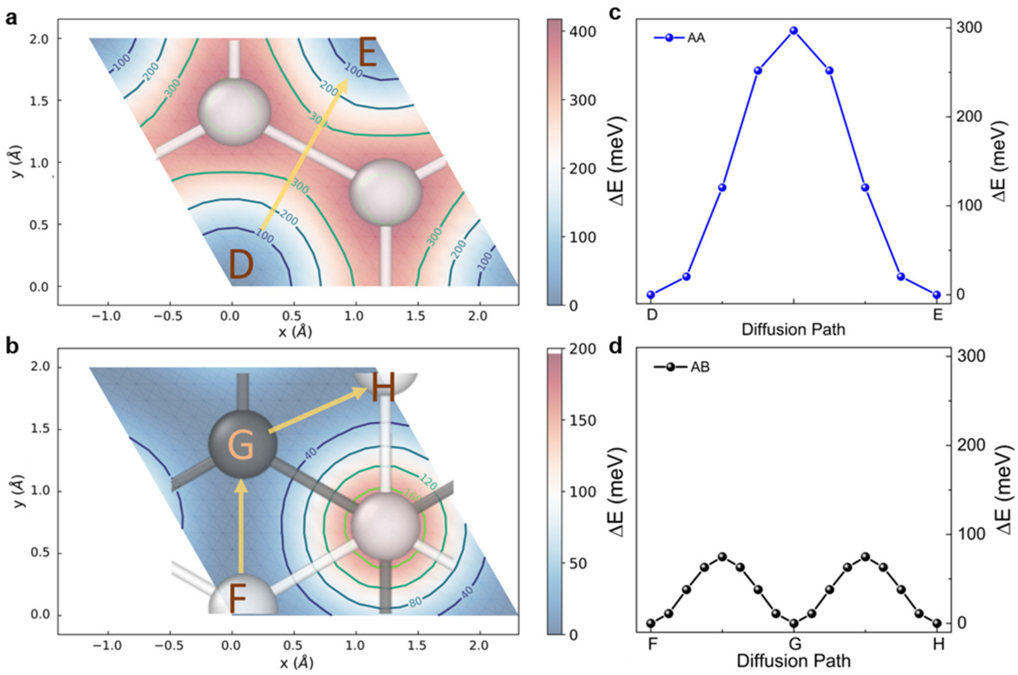

Supplementary Figure 10 | Nudged elastic band (NEB) barriers for a Li atom diffusing in AA- & AB-stacked bilayer graphene. (a,b) Energy landscape for Li-intercalated AA- and AB- stacked bilayers. The arrows show optimal diffusion paths for Li ion jumps by one lattice constant via saddle points in the energy maps. **(c,d)** Corresponding activation energies. In AB-stacked bilayer, Li ions can entirely avoid the large maximum associated with the interlayer C-C dimer.

2.4. Screening of interionic interactions.

The importance of electrostatic repulsion between intercalated ions in layered materials, where ions intercalate into interlayer galleries, has long been appreciated, notably for graphite [24-27], MXenes [28] and TiS_2 [29]. The authors of refs. [30, 31] used a simple model of point charges interacting through screened electrostatic interactions to argue that screening has a strong effect on attainable intercalation densities. This effect is especially important for intercalation of ions into BLG, where the same planar density of Li ions per carbon layer (C_xLiC_x) as in bulk graphite (LiC_x) results in less Li-dense configurations overall, simply because the same number of Li ions are shared by the two adjacent layers in BLG but not in bulk graphite. As the result, the host carbon atoms are less strongly doped, because the amount of charge lost by one Li ion to the surrounding graphene layers is approximately the same in both cases. Accordingly, the density of charge carriers at the Fermi level in Li-intercalated BLG is lower, which in turn leads to relatively ineffective screening of the interionic repulsion.

Our DFT calculations incorporate the effect of screening naturally and allow numerical estimation of its impact. We find a very strong effect of the poorly screened Coulomb repulsion on ionic mobility, as shown in Supplementary Fig. 11 through comparison of off-stoichiometric arrangements of Li ions in a repeated

$\sqrt{7} \times \sqrt{7}$ superlattice. It demonstrates a significantly higher energetic penalty in the bilayer case, in agreement with the qualitative arguments above.

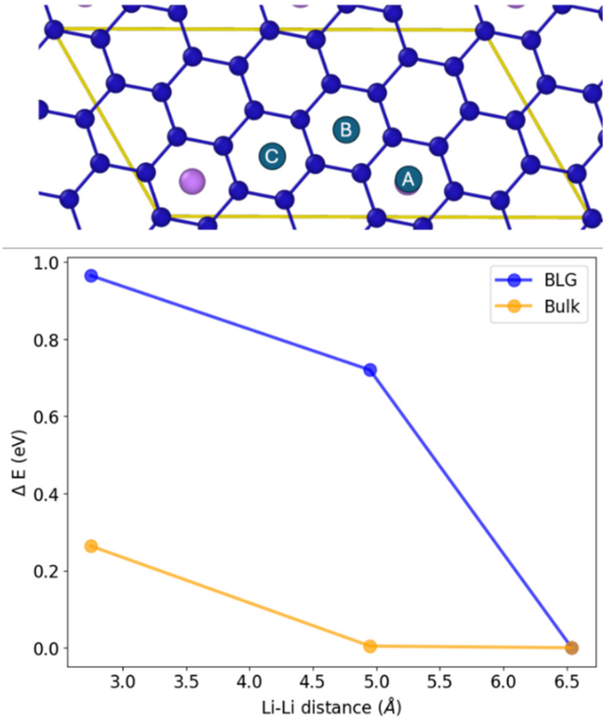

Supplementary Figure 11 | Comparison of the energy penalty for off-stoichiometric arrangements of Li-ions in bulk graphite and bilayer graphene. *Top panel*: Three distinct structures considered in the calculations. Each structure contains two Li ions but the distances between the two ions are different in each case. For structure (A) the position of the second Li ion corresponds to the experimentally found stoichiometry, with Li-Li distance ≈ 6.5 Å. For (B) and (C) the interionic distance is artificially decreased in order to quantify the Coulomb repulsion. *Bottom panel*: Increase in the interaction energy (referenced to the experimental stoichiometry) as a function of interionic distance for bulk graphite (yellow symbols) and for BLG (blue). The energy penalty is markedly higher for the bilayer.

Supplementary References

1. Méry, A., Rousselot, S., Lepage, D., Dollé, M. A critical review for an accurate electrochemical stability window measurement of solid polymer and composite electrolytes. *Materials* **14,** 3840 (2021).
2. Chen, Y. et al. Nanoarchitecture factors of solid electrolyte interphase formation via 3D nano-rheology microscopy and surface force-distance spectroscopy. *Nat. Commun.* **14**, 1321 (2023).
3. Yun, J. M. et al. Complementary p- and n-type polymer doping for ambient stable graphene inverter. *ACS Nano* **8**, 650–656 (2014).
4. Pirkle A, et al. The effect of chemical residues on the physical and electrical properties of chemical vapor deposited graphene transferred to SiO_2. *Appl. Phys. Lett.* **99**, 122108 (2011).
5. Levi, M. D., Markevich, E., Aurbach, D. The effect of slow interfacial kinetics on the chronoamperometric response of composite lithiated graphite electrodes and on the calculation of the chemical diffusion coefficient of Li ions in graphite. *J. Phys. Chem. B* **109**, 15, 7420-7427 (2005).

6. Cancado, L. G. et al. Quantifying defects in graphene via Raman spectroscopy at different excitation energies. *Nano Lett.* **11**, 3190—3196 (2011).
7. Giannozzi, P. et al. Quantum ESPRESSO: a modular and open-source software project for quantum simulations of materials. *J. Phys.: Condens. Matter* **21**, 395502 (2009).
8. Giannozzi, P. et al. Advanced capabilities for materials modelling with Quantum ESPRESSO. *J. Phys.: Condens. Matter* **29**, 465901 (2017).
9. Garrity, K. F., Bennett, J. W., Rabe, K. M., Vanderbilt, D. Pseudopotentials for high-throughput DFT calculations, *Computational Materials Science* **81**, 446-452 (2014).
10. Perdew, J. P., Burke, K., Ernzerhof, M. Generalized gradient approximation made simple. *Phys. Rev. Lett.* **77**, 3865-3868 (1996).
11. Monkhorst, H. J. and Pack, J. D. Special points for Brillouin-zone integrations. *Phys. Rev. B* **13**, 5188-5192 (1976).
12. Klimeš, J., Bowler, D. R., & Michaelides, A. Chemical accuracy for the van der Waals density functional. *J. Phys.: Conden. Matter* **22**, 022201 (2009)
13. Hazrati, E., de Wijs, G. A., & Brocks, G. Li intercalation in graphite: A van der Waals density-functional study. *Phys. Rev. B* **90**, 155448 (2014).
14. Yang, C.-K. A metallic graphene layer adsorbed with lithium. *Appl. Phys. Lett.* **94**, 163115 (2009).
15. Sutton, C., Levchenko, S. V. First-principles atomistic thermodynamics and configurational entropy. *Front. Chem.* **8**, 757 (2020).
16. Reynier, Y. F., Yazami, R., & Fultz, B. (2004). Thermodynamics of lithium intercalation into graphites and disordered carbons. *J. Electrochem. Soc.* **151**, A422 (2004).
17. Dai, S., Xiang, Y., Srolovitz, D. J. Structure and energetics of interlayer dislocations in bilayer graphene. *Phys. Rev. B* **93**, 085410 (2016).
18. McHugh, J. G., Mouratidis, P., Jolley, K. Ripplocations in layered materials: Sublinear scaling and basal climb. *Phys. Rev. B* **103**, 195436 (2021).
19. Plimpton, S. Fast parallel algorithms for short-range molecular dynamics, *J. Comp. Phys.* **117**, 1-19 (1995).
20. Brenner, D. W., Shenderova, O. A., Harrison, J. A., Stuart, S. J., Ni, B., Sinnott, S. B. A second-generation reactive empirical bond order (REBO) potential energy expression for hydrocarbons. *J. Phys.: Condens. Matter*, **14**, 783-802 (2002).
21. Wen, M., Carr, S., Fang, S., Kaxiras, E., & Tadmor, E. B. Dihedral-angle-corrected registry-dependent interlayer potential for multilayer graphene structures. *Phys. Rev. B* **98**, 235404 (2018).
22. Maslov, M. M., Openov, L. A., & Podlivaev, A. I. On the vineyard formula for the pre-exponential factor in the Arrhenius law. *Physics of the Solid State* **56**, 1239–1244 (2014).
23. McHugh, J. G., Jolley, K. & Mouratidis, P. Ab-initio calculations of fission product diffusion on graphene. J. Nuclear Mater. **533**, 152123 (2020).
24. Allart, D., Montaru, M., Gualous, H. Model of lithium intercalation into graphite by potentiometric analysis with equilibrium and entropy change curves of graphite electrode. *J. Electrochem. Soc.* **165**, A380 (2018).
25. Anniés, S. et al. Accessing structural, electronic, transport and mesoscale properties of Li-GICs via a complete DFTB model with machine-learned repulsion potential. *Materials* **14**, 6633 (2021).
26. Panosetti, C., Annies, S. B., Grosu, C., Seidlmayer, S., Scheurer, C. DFTB modeling of lithium-intercalated graphite with machine-learned repulsive potential. *J. Phys. Chem. A* **125**, 691–699 (2021).

27. Pande. V. and Viswanathan, V. Robust high-fidelity DFT study of the lithium-graphite phase diagram. *Phys. Rev. Materials* **2**, 125401 (2018).

28. Eames, C. & Saiful Islam, M. Ion intercalation into two-dimensional transition-metal carbides: Global screening for new high-capacity battery materials. *J. Am. Chem. Soc.* **136**, 16270–16276 (2014).

29. Jacobsen, T., West, K., Atlung, S. Electrostatic interactions during the intercalation of Li in Li_xTiS_2. *Electrochimica Acta* **27**, 1007-1011 (1982).

30. DiVincenzo, D. P., Mele, E. J. Electrostatic effects in the cohesion of an intercalant lattice. *Phys. Rev. B* **25**, 7822-7825 (1982).

31. DiVincenzo, D. P., Mele, E. J. Self-consistent effective-mass theory for intralayer screening in graphite intercalation compounds. *Phys. Rev. B* **29**, 1685–1694 (1984).
